# A Flexible Hierarchical Framework for Implicit 3D Characterization of Bionic Devices

**DOI:** 10.3390/biomimetics9100590

**Published:** 2024-09-29

**Authors:** Yunhong Lu, Xiangnan Li, Mingliang Li

**Affiliations:** 1 School of Computer and Control Engineering, Yantai University, Yantai 264005, China; luyunhong@ytu.edu.cn (Y.L.); limingliang@s.ytu.edu.cn (M.L.); 2 Yantai Science and Technology Innovation Promotion Center, Yantai 264005, China

**Keywords:** bionic equipment, multi-view, 3D model reconstruction, hierarchical octree, adaptive mechanism

## Abstract

In practical applications, integrating three-dimensional models of bionic devices with simulation systems can predict their behavior and performance under various operating conditions, providing a basis for subsequent engineering optimization and improvements. This study proposes a framework for characterizing three-dimensional models of objects, focusing on extracting 3D structures and generating high-quality 3D models. The core concept involves obtaining the density output of the model from multiple images to enable adaptive boundary surface detection. The framework employs a hierarchical octree structure to partition the 3D space based on surface and geometric complexity. This approach includes recursive encoding and decoding of the octree structure and surface geometry, ultimately leading to the reconstruction of the 3D model. The framework has been validated through a series of experiments, yielding positive results.

## 1. Introduction

Biomimetics is an interdisciplinary research field that addresses complex engineering and design challenges by mimicking principles and structures found in nature. In recent years, significant advancements have been made in biomimetics across various domains, including advanced medical devices and robotics. These advancements highlight the immense potential of biomimetics for innovative design. For example, recent studies have demonstrated drones that emulate bird flight mechanisms [1], climbing robots that mimic the adhesive properties of gecko feet [2], and waterproof materials inspired by the lotus leaf effect [3]. These technological innovations emphasize the broad application prospects of biomimetics but also raise higher demands for the design and optimization of such devices.

Effective design and optimization of bionic devices require precise and accurate characterization of their three-dimensional features. Three-dimensional characterization technologies play a crucial role in this process by providing detailed modeling of complex structures and functions, which is essential for accurate simulation and performance enhancement of the devices. Despite the foundational progress made by traditional 3D characterization methods in capturing geometric details and functional characteristics, these methods often face significant challenges as the complexity of bionic devices increases.

This study introduces a novel 3D characterization framework aimed at addressing the shortcomings of traditional methods in handling complex biomimetic structures. The framework improves the precise description of biomimetic structures by offering high-resolution, adaptive modeling techniques. Specifically, this work integrates state-of-the-art 3D reconstruction technologies, including adaptive local support region segmentation based on sparse voxel octrees, recursive encoding and decoding methods, to enhance the design and performance evaluation of bionic devices [4,5,6,7,8]. Through these technologies, this study bridges the gap between biomimetic innovations and practical applications, providing a solid foundation for the engineering optimization of bionic devices.

From scene understanding and object recognition to 3D shape reconstruction and virtual reality scene reconstruction, 3D representation of geometric surfaces has always been at the core of computer vision and computer graphics tasks. However, there is inherent uncertainty in the 3D representation of geometric surfaces in 3D shape reconstruction. Inefficiency exists in a range of objects with irregular geometries and surfaces, such as images of human bodies and animals. Based on this, aiming at voxel-based 3D reconstruction, high-quality reconstruction of objects with a large number of irregular geometries and surfaces can be achieved.

Explicit representation methods, such as mesh generation and finite element analysis, rely on precise geometric descriptions. These methods offer high accuracy when dealing with regular geometries; however, they often require high-density meshes to capture the details of complex biomimetic structures, leading to significantly increased computational costs. Additionally, explicit methods have limitations in handling shape changes and topological transformations.Implicit representation methods, such as distance fields and level set methods, provide a means of describing geometries through implicit functions. These methods offer significant advantages in handling complex shapes and topological changes. However, implicit methods must strike a balance between achieving high-precision descriptions and maintaining computational efficiency.

Although explicit 3D representation is widely used in 3D reconstruction work, there is no existing work that can satisfy the corresponding properties of the method. The challenge of preserving fine-scale shape details is particularly problematic for point cloud-based and voxel-based representations, because doing so frequently requires considerable memory consumption. Mesh-based learning methods tend to rely on the deformation of the model, thus limiting the scalability of this class of methods to handle arbitrary topologies. The neural implicit network [4,9,10,11,12] suggested by Chen et al. offers a superior strategy for 3D modeling and reconstruction challenges. However, the use of global functions to code all shapes in neural implicit networks reduces the reconstruction accuracy and shape applicability of the model.

To circumvent these challenges, it has been suggested that three-dimensional spaces be transformed into lattices [5,13] or local support spaces [14], with the geometry of each space being provided to a local implicit function for approximation expression.The meshing of the space increases the precision and effectiveness of the restructuring process while also enhancing the shape prior that each local network must learn. However, these methods do not account for the fact that the granularity of local geometric surface representations varies, resulting in two issues. First, memory utilization increases quadratically with the volume of the 3D scene; second, standard meshing is difficult to scale to high resolution, limiting the representability of geometric surfaces when dealing with complicated, fine-grained, dense structures.

By evaluating a large number of 3D form representations, it is possible to deduce that geometric surfaces often contain a greater number of smooth surfaces and fewer fine details. At the same time, the reconstructed surface usually occupies only a small part of the total space. Therefore, the Sea-OctField 3D surface representation method is proposed in this paper, which introduces the hierarchical structure into the organization of local implicit functions to improve the efficiency of 3D reconstruction.

However, octrees have a distinct and infinitesimal structure, and thus, it is essential to employ them directly in a deep learning framework. In this research, a novel hierarchical network is proposed for distinguishing recursive encoding and decoding of octree structures and geometric features. On the decoder side, the subdivision of octree cells is specifically clearly stated as a probabilistic process, which requires learning the octree structure microscopic. In addition, a classifier is utilized in this method to determine whether the current cell should be subdivided based on its closed geometric properties.

As technology continues to advance, bionic devices are playing an increasingly important role in fields such as medicine, robotics, and bioengineering. These devices are often designed to mimic or enhance natural biological structures and functions to help restore or replace lost physiological functions (for example, the bionic hand). However, the design and manufacture of bionic devices faces many challenges, especially in scenarios that require a high degree of personalisation and precise adaptation. Traditional design methods are often difficult to meet the requirements of complex morphology and high precision, limiting the widespread use of bionic devices in practical applications. Three-dimensional reconstruction technology is capable of accurately capturing and reconstructing complex biological structures, providing a solid foundation for the personalised design and manufacture of bionic devices. Although 3D reconstruction technology shows great potential in the design and manufacture of bionic devices, there are still some challenges in practical applications. How to improve the reconstruction accuracy, especially in the reconstruction of complex tissues or dynamic structures, requires more efficient algorithms and more powerful computational capabilities.

**Figure 1:**
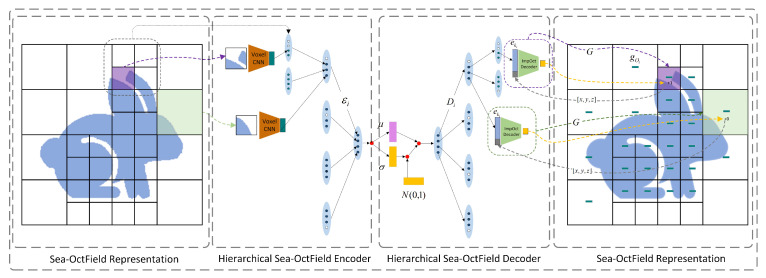
A diagram of a hierarchical octree neural network in two dimensions. In this paper, a recursive encoder–decoder network is proposed, which is trained using several GAN methods. Here, the geometry of the octree is encoded using the voxel 3DCNN and recursively aggregated using the hierarchical structure and geometric features of the local encoder $\varepsilon_{i}$. The decoding function is implemented by a local decoder $D_{i}$ hierarchy with a mirror structure relative to the encoder. The structural and geometric information of the input model is decoded recursively, and the local geometric surfaces are recovered with input of an implicit decoder embedded in each octree.

Mesh-based methods usually lack geometric expansion performance and do not take into account the memory bottleneck caused by the granularity differentiation of different geometric details. NeRF-based methods have unique features that are difficult to discriminate when dealing with regions with high texture repetition or sparse texture, which may lead to repetition errors and unstable model performance. The inability to effectively distinguish features in these regions results in errors that affect the overall reliability of the reconstruction. Methods based on generative approaches are highly dependent on the quality of the dataset. To address the above issues, we adopt the recursive encoder–decoder network, as shown in Figure 1, which is trained using various GAN methods. In this work, voxel 3DCNN is used to encode the geometry of the octree and recursive aggregation is performed using the hierarchy of local encoders and geometric features. The decoding function is implemented by a hierarchy of local decoders with a mirror structure relative to the encoder that recursively decodes the structural and geometric information of the input model. Moreover, the local geometric surface is recovered by embedding the input of an implicit decoder in each octree. The following three primary points constitute the key contributions of this paper:

- This work is able to extract the density structure of 3D objects from multiple views, resulting in a higher quality 3D structure compared to traditional point cloud data.
- This work provides high-quality reconstruction of complex geometries by introducing a new neural network layer.
- This work solves the distortion problem in 3D model reconstruction work in geometric scenes by improving the OctField algorithm.

## 2. Related Work

Representation of three-dimensional forms based on deep learning. Diverse 3D form representations are gaining increasing attention and research in the field of 3D deep learning [15]. The survey by Eman Ahmed et al. [16,17] gives a thorough analysis of available deep learning-based 3D form representation techniques. In recent years, point clouds have gained considerable attention [18]. As the output of 3D scanning equipment, point cloud data makes it simple to collect 3D models with high precision, yet it remains a formidable task to build dense point cloud models with high precision [19]. Contrary to conventional representations of 3D geometric surfaces, convolutional neural networks can process 3D voxels [20,21]. Currently, the computational cost of voxel-based generative models is significant [22], and new research has added the notion of octree space to the geometric representation of voxels in order to lower the storage cost [23]. Also, meshes are frequently used to represent 3D surfaces in 3D model reconstruction tasks. Ding et al. [24] proposed a bionic active sensing algorithm for 3D perception and reconstruction in order to improve the visual perception accuracy as well as the control of force and position of a bionic robot, which achieves high-precision 3D modeling by applying a registration algorithm.

However, current mesh-based methods [25,26,27,28] for producing 3D models enhancing accuracy primarily by deforming the mesh, restricting its scalability to shapes with arbitrary topology. Because of the adaptability of the neural implicit function [4,5,29] in dealing with arbitrary topology of the mesh surface, reconstruction of the mesh surface by the neural implicit function can improve the accuracy of the 3D model surface reconstruction works. By locally recreating geometric characteristics, Kyle Genova et al. [5,13,14] added shape decomposition and local implicit functions to the model to boost its modeling capability. Presenting the discrete dot set of the Implicit Moving Least Squares (IMLS) [23] surface-resolution model, Shi-Lin Liu et al. defined high-grade geometric zones. Existing approaches, however, focus primarily on a linear decomposition of the geometric space and do not account for the occupancy of sparse geometric surfaces and the variable granularity of geometric details, which might result in memory bottlenecks when partitioning moderately dense geometric surfaces.

Recently, NeRF-based methods have been developed to generalize networks for cross-scene training for efficient 3D reconstruction of small amounts [30]. Chen et al. [31] proposed a novel neural rendering method known as MVSNeRF, which is capable of effectively reconstructing neural radiance fields for view synthesis and representing objects within three-dimensional scenes. This approach utilizes rapid network inference to reconstruct the radiance field from three nearby input views. Subsequently, it employs plane-sweeping cost volumes for geometry-aware scene inference, combining this with physically based volumetric rendering to achieve the reconstruction of the neural radiance field. MVSNeRF excels in providing three-dimensional representations of input images across different scenes, generating highly realistic synthesized results. Furthermore, the method allows for fine-tuning of the scene’s radiance field, enabling swift reconstruction of scenes. This capability is particularly beneficial for applications requiring quick and accurate 3D reconstructions, such as virtual reality, augmented reality, and various fields of computer vision. Despite its advantages, the method encounters challenges when dealing with regions that exhibit high texture repetition or areas with sparse texture. In such regions, the distinctive features are difficult to discern, which may lead to repeated errors and instability in the model’s performance. The inability to effectively differentiate features in these areas results in inaccuracies, affecting the overall reliability of the reconstruction.

Generative models utilizing deep learning. The ability with deep generative models, along with the two deep generative models GAN [32] and VAE [33], to obtain realistic images in the 2D domain has been consistently proven, but the ability to produce high-quality 3D models in the 3D domain has managed to gain more attention. Three-dimensional learning approaches strive to replicate 2D generative models successfully in order to generate 3D shapes. The 3D-GAN [26] model initially employed GAN techniques to voxels to discover a deep generator capable of synthesizing multiple 3D shapes. Although it is true that MLP layers are frequently implemented in generative models on point clouds [29], it might be difficult to build dense point sets with high precision because to computational resources and computational complexity. Recent work on synthetic 3D meshes mainly relies on graph-CNN [10,25,34] to deform mesh shapes or manipulate structures by assembling surfaces [5,35]. A hierarchical implicit generative model for 3D modeling was introduced by OctField [6], and it was used to reconstruct 3D surfaces with fantastic quality and intricate geometric elements.

Ho et al. [36] proposed a cascading diffusion model capable of generating high-fidelity images without the aid of auxiliary image classifiers. This cascading diffusion model is structured as a pipeline composed of multiple diffusion models, each tasked with generating images of progressively higher resolutions. The methodology enhances image information by incrementally adding higher resolution details through upsampling. This approach is particularly effective in extracting fine details within the images. However, it is noteworthy that as the number of cascading layers increases, the computational cost correspondingly escalates. Moreover, the cascading diffusion model imposes stringent requirements on the quality and completeness of the input data.

Bautista et al. [37] proposed a GAUID generative model capable of capturing the distribution of complex and realistic 3D scenes, which can be rendered immersively by moving the camera. This method optimizes a latent 3D representation to disentangle the radiance field from the camera poses. By learning this latent 3D representation, the generative model can both unconditionally and conditionally generate 3D scenes. The model employs a sophisticated neural network architecture to effectively learn the underlying structure and appearance of 3D environments, providing high-quality visual outputs. However, the model exhibits strong performance on specific datasets but demonstrates decreased generalization ability when applied to different types of data. This limitation means that the model might not perform as well when faced with new, unseen environments that differ significantly from the training data. Additionally, there is a significant risk of overfitting during the training process.

Henzler et al. [38] proposed a method called PlatonicGAN, which discovers the 3D structure of object classes from unstructured 2D image collections. The key idea of this method is to train a deep neural network to generate 3D shapes. This method employs a series of distinguishable rendering layers to establish constraints between the 2D image observations and their 3D interpretations, enabling the reconstruction of 3D shapes from unstructured 2D images. The approach leverages the power of deep learning to infer 3D structures by learning from large datasets of 2D images, effectively bridging the gap between 2D observations and 3D shape generation. However, this method heavily relies on the quality and diversity of the training data. If the training data contain noise, biases, or insufficient variety, the model may struggle to generalize well to new, unseen data, leading to a significant risk of overfitting.

Watson et al. [39] proposed a diffusion model called 3DiM for 3D view synthesis, which can transform a single input view into multiple views. The core of this method is a pose-conditional image-to-image diffusion model that takes the source view and its pose as input, and generates new views for the target poses in an autoregressive manner, outputting these as the final result. Generating high-quality multi-view consistent images and 3D shapes from a collection of single-view 2D photos in an unsupervised manner remains a significant challenge in computer vision. Existing methods often face a trade-off between the quality and resolution of generated images and the preservation of multi-view consistency or the accuracy of 3D shapes. In their work on 3DGAN, Chan et al. [40] proposed a novel hybrid explicit–implicit network architecture. This architecture combines the advantages of explicit representations, which offer precise control over 3D shapes, with implicit representations, providing flexibility to model complex structures. By leveraging this hybrid approach, 3DGAN achieves real-time synthesis of high-resolution, multi-view consistent images and generates high-quality 3D geometries, which is a significant advancement in the field. However, the effectiveness of 3DGAN is contingent upon the quality of the input images. High-quality input data are crucial for ensuring fidelity in the synthesized views and accuracy in the reconstructed 3D shapes. Moreover, as with many deep learning models, 3DGAN is susceptible to overfitting during training, which can limit its generalization ability to unseen data. Addressing these challenges requires ongoing research efforts to improve the robustness, generalization, and overall performance of 3DGAN. Enhancements in data preprocessing techniques, regularization strategies, and model architectures are essential to further advance the capabilities of unsupervised 3D shape synthesis from single-view images.

In the work of this paper, the generation of high-quality 3D structures is done using a periodic implicitly generated adversarial network (pi-GAN) [41]. pi-GAN senses image generation unconditionally and is able to reconstruct 3D shapes and textures conditionally from local observations. Using pi-GAN to generate 3D structures will allow us to generate models unconditionally.

## 3. Method

This section focuses on the eight-domain hierarchical network and the deep generative model mentioned in this work.

### 3.1. Hierarchical Eight-Domain Network

Octree structure. In integrating the input model into the octree structure segmentation, the 3D structure is first scaled evenly into symmetric bounding boxes, followed by recursive subdivision of the bounding regions into suboctrees in breadth-first order. In order to subdivide a model, two conditions must be met. The surface of interest is contained within the octree, and the surrounding geometry must be sufficiently complex to justify subdivision. Specifically, the following formula describes the normal variance of a surface slice *S*:

(1) $$\nu \left(S\right)=E_{i}\left(\nu \left(n_{x}^{i}\right)+\nu \left(n_{y}^{i}\right)+\nu \left(n_{z}^{i}\right)\right),$$

where $n_{x}^{i}$, $n_{y}^{i}$, and $n_{z}^{i}$ are *x*, *y*, and *z* elements, respectively, that make up the normal vector $n^{i}$ at the *i*-th surface point that was sampled, $n_{x}^{i}$ denotes the set of $n_{x}^{i}$; ν(·) calculates the variation of the input, while $E_{i}\left(\cdot \right)$ returns the expected value. In the test, periodic sampling is performed on the surface, and the sampling points are precomputed. The decomposition is repeated until predefined depth *d* or ν(S) less than predefined threshold τ is reached.

(2) $$\left(e_{c_{0}},e_{c_{1}},\cdots ,e_{c_{7}}\right)=D_{k}\left(g_{k}\right)$$

where $c_{j}\in C_{k}$ represents the subtree of $O_{k}$ and $e_{c_{j}}=\left(g_{c_{j}},\alpha_{c_{j}},\beta_{c_{j}}\right)$ represents the geometric properties of the subtree $O_{c_{j}}$, as well as two metrics.These two metrics indicate the likelihood that subtree must be decoded or partitioned. This is achieved by decoding all eight suboctets simultaneously.

**Figure 2:**
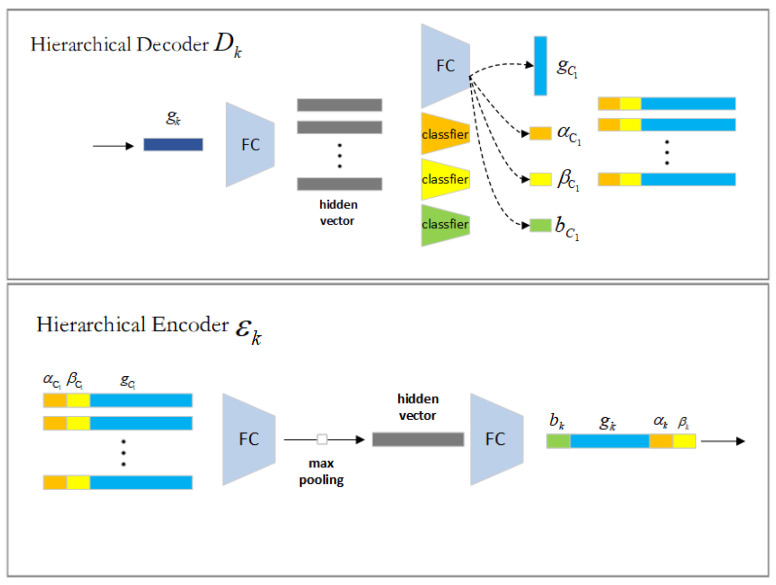
The structure of the encoder and decoder $E_{k}$ and $D_{k}$, respectively. $E_{k}$ collects the structure ($a_{c_{j}},b_{c_{j}}$) and geometric characteristics ($g_{c_{j}}$) of the child octrees into its parent octree *k*, where $c_{j}$ is in $C_{k}$, utilizing an MLP, maximum set operation, and second MLP. Two MLPs and classifiers decode the geometric features $g_{k}$ of the parent space into geometric features $g_{c_{j}}$ and two attributes $\alpha_{c_{j}}$,$\beta_{c_{j}}$ of the child space. Two metrics are employed to determine the probability of surface occupation and the need for substructure subdivision.

As seen in Figure 2, the encoding process begins from the bottom up with the most significant octet. For each octagon $O_{i}$, its binary metric $\left(\alpha_{i},\beta_{i}\right)$ is first calculated based on its closed geometry. If surface exists inside $O_{i}$, $\alpha_{i}$ is set to 1, otherwise it is 0. If the closed geometry of $O_{i}$ (if $\alpha_{i}$ = 1) satisfies the subdivision criterion, $\beta_{i}$ is set to 1. Then, its closed voxelized geometry $G_{i}$ is passed to the voxel CNN *V* to extract the geometric features $G_{i}$ of $O_{i}$. When proceeding to higher levels, the potential features of the children are aggregated to their parent octants. In particular, for the parent octet $O_{K}$, this method represents the features of its child octets as $e_{c_{j}}=\left(b_{c_{j}},g_{c_{j}},\alpha_{c_{j}},\beta_{c_{j}}\right)|c_{j}\in C_{k}$, where represents the child octet of $O_{k}$. The latent features of the substrate of the encoder $\varepsilon_{k}$ of $O_{i}$ to the geometric feature $g_{k}=\varepsilon_{k}\left(e_{c_{0}},e_{c_{1}},\cdots ,e_{c_{7}}\right)$ of $O_{i}$. Then, $g_{k}$ is connected with the structural features $\left(\alpha_{k},\beta_{k}\right)$ of $O_{K}$ to obtain the latent features of $O_{i}$. Until the root node is processed, the encoding and fusing of recursive geometric features are accomplished.

$D_{k}$ comprises specifically two MLPs and three classifiers. First, $g_{k}$ is decoded by one MLP into hidden vector $v_{c_{j}}$ for all eight subspaces. For this method, two classifiers $L_{g}$ and $L_{h}$ are used to retrieve model structure information to compute the probability of the surface being occupied and, respectively, the necessity for further segmentation. For the subspace $O_{c_{j}}$, its hidden vector $v_{c_{j}}$ is input to $L_{g}$ and $L_{h}$ and $\alpha_{c_{j}}=L_{g}\left(v_{c_{j}}\right)$, $\beta_{c_{j}}=L_{g}\left(v_{c_{j}}\right)$ and $b_{c_{j}}=L_{h}\left(v_{c_{j}}\right)$ are computed. To predict $g_{c_{j}}$, other MLPs are applied on $v_{c_{j}}$. Then, it means that $O_{c_{j}}$ does not contain any geometry and will not be processed further. If $\alpha_{c_{j}}\leq 0.5$, it indicates that $O_{c_{j}}$ does not contain any geometry and is not processed further. If $\alpha_{c_{j}}>0.5$, indicating that $O_{c_{j}}$ is occupied by surfaces and the value of $\beta_{c_{j}}$ will be checked further. If $\beta_{c_{j}}\leq 0.5$, the method will not further subdivide the octagon and will use the implicit octagon decoder *G* and the geometric feature $g_{c_{j}}$ to infer the surface it encloses. If $\beta_{c_{j}}>0.5$, the space will be subdivided by the same procedure by predicting the latent features of its subnodes. $b_{c_{j}}$ represents the decoding recursion depth generated by the encoder. We repeat this procedure until there is no space to be subdivided.

### 3.2. Deep Generation Model

The deep generator model uses a generative method for learning radiation field representations from unlabeled 2D images to generate high-quality 3D density sets, a type of data similar to point clouds.

**Figure 3:**
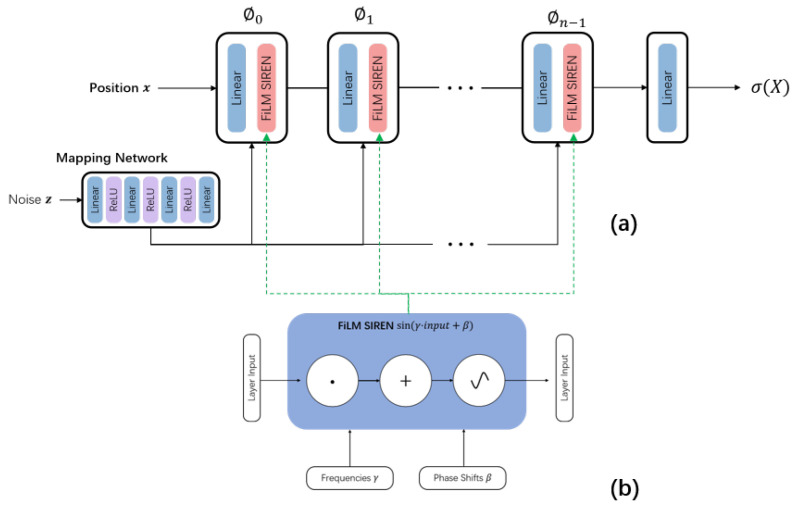
This figure shows the depth generation model, which is composed of *n* combined network layers of linear network and implicit network, and the implicit network core uses sin function as the calculation method. The multi-layer perceptron takes the position information *x* and the noise information *z* processed by the mapping network layer as the input, and the final output is the density. (**a**) Overall architecture of the network model. (**b**) Specific structure of the FiLM SIREN unit.

Three-dimensional objects are implicitly represented in the generator using a neural radiation field parameterized by a multilayer perceptron (MLP), which takes as input the space x=(x,y,z) and the three-dimensional coordinates *d* in the observation direction. The output of the neural radiation field varies in space with a density of σ(x), as shown in Figure 3a.

The neural radiation field output spatially varies in density $\sigma \left(x\right):R^{3}\to R$. In addition, we employ a mapping network inspired by StyleGAN [42] to alter the siren based on the noise vector *z* via film conditioning. As represented in Figure 3a, the generator is represented as:

(3) $$\phi \left(x\right)=\phi_{n-1}\circ \phi_{n-2}\circ \cdots \circ \phi_{0}\left(x\right),$$

(4) $$\phi_{i}\left(x_{i}\right)=\sin(\gamma_{i}\cdot \left(W_{i}x_{i}+b_{i}\right)+\beta_{i},$$

where $\phi_{i}:R^{M_{i}}\mapsto R^{N_{i}}$ is the *i*-th layer of the MLP. The operator ∘ represents the function composition used to build network models. It consists of an affine transformation defined by the weight matrix $W_{i}\in R^{N_{i}\times M_{i}}$ and a bias $b_{i}\in R^{N_{i}}$ applied to the input $x_{i}\in R^{M_{i}}$; it is then utilized as a nonlinear sine wave for each resultant vector component (Figure 3b). The network utilized in this instance is a basic ReLU MLP that modulates each layer of alerts by receiving the noise vector *z* as input and outputting the frequency $\gamma_{i}$ and phase shift $\beta_{i}$ for that item. The density representation of the implicit function is then characterized as follows:

(5) $$\sigma \left(x\right)=W_{\sigma }\phi \left(x\right)+b_{\sigma }$$

where $W_{\sigma }$ and $b_{\sigma }$ are additional weights and deviation parameters.

## 4. Experiment

In this section, the data preparation methodology for the experiments will be described first, followed by an evaluation of the proposed method in several applications, such as shape reconstruction, form production, interpolation, and shape completeness.

### 4.1. Shape Reconstruction

**Figure 4:**
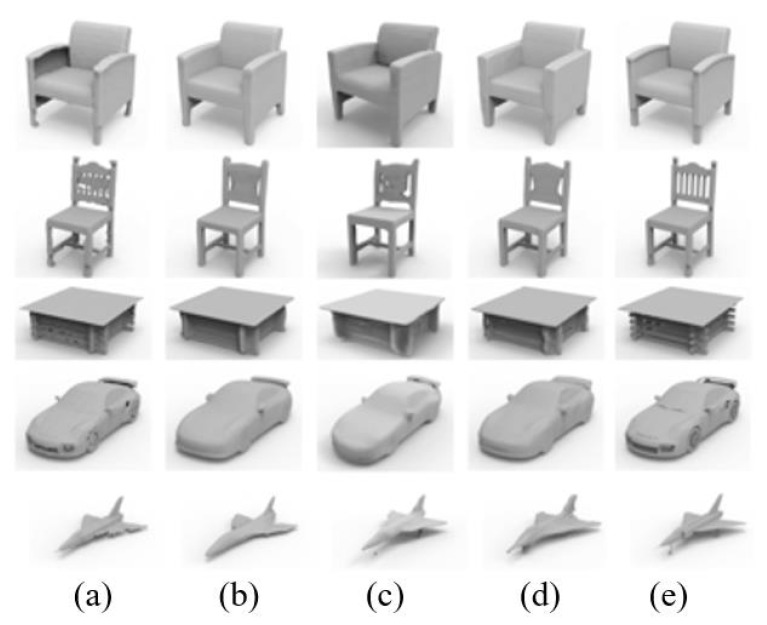
Shape reconstruction comparison of (**a**) LIG [5], (**b**) OccNet [9], (**c**) IM-Net [4], (**d**) OctField [6], and (**e**) the work of this paper.

The ShapeNet dataset’s [43] five largest and most prevalent object classes were employed in order to train and evaluate a dataset used to evaluate the performance of shape reconstruction in this section: chairs, tables, airplanes, cars, and sofas. The most recent technologies that are closely related to those utilized in this research are also used for comparison, and these are:IM-Net [4], OccNet [44], Locally Implicit Grid (LIG) [5], and OctField [6]. While LIG classifies the input model into grid data, uses linear rules, and uses a local implicit kernel to complete the geometric representation of the model, OctField and OccNet both use implicit functions to complete the 3D shape representation. Moreover, while IM Net and OccNet can recreate the overall form of an object, they are unable to do so for its intricate structure. LIG could recover some fine geometry, but it struggles to reproduce the angles and delicate structures shown in the second row. In comparison, the method developed in this paper gets better results across all categories (see Figure 4 for the control findings) and is capable of reconstructing complicated geometric details such as the slats of a chair back, the skeletonized base of a table, and the wheels of an automobile.

The evaluation metrics used in this section and their functions are as follows:

- **Mean Intersection over Union (mIoU)**: Measures the overlap between predicted and ground truth shapes by averaging the ratio of the intersection area to the union area of the predicted and ground truth shapes.
- **F1 Score**: The harmonic mean of precision and recall. Precision is the proportion of true positive predictions out of all positive predictions, while recall is the proportion of true positives out of all actual positives.
- **Chamfer Distance (CD)**: Calculates the distance between point clouds of the predicted and ground truth shapes by averaging the distance from each point to its nearest neighbor.
- **Earth Mover’s Distance (EMD)**: Also known as Wasserstein distance, it measures the difference between two point cloud distributions by calculating the minimum amount of work required to move one distribution to the other.

mIoU is used to measure the overlap between the model’s predictions and the actual shapes, reflecting the model’s accuracy. F1 is used to evaluate the model’s precision and recall comprehensively, assessing the overall performance of the model. CD is used to measure the geometric differences between the predicted and ground truth shapes, reflecting the model’s precision. EMD is used to assess the similarity of shape distributions, suitable for measuring the gap between generated shapes and actual shapes. For all metrics, 10 experiments were conducted in ShapeNet dataset and the optimal values were taken as the results.

**Figure 5:**
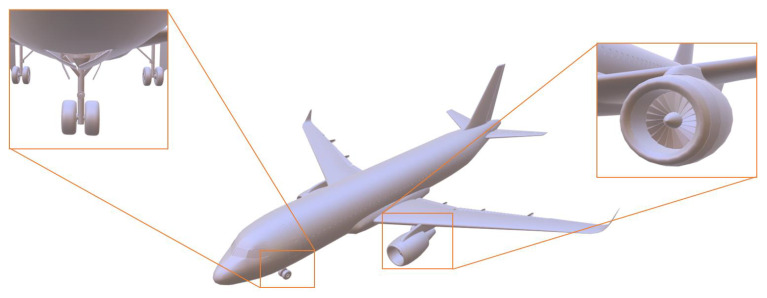
The result of modeling the detail part of the aircraft model.

Figure 5 depicts the reconstruction results of the method suggested in this paper for the complicated surfaces of the model.

Based on the content of the two tables, the proposed method in this paper significantly outperforms other comparative methods in the shape reconstruction task. Table 1 shows the mIoU and F1 scores, where the proposed method achieves an mIoU of 88.32 and an F1 score of 0.95, the highest among all methods. In contrast, OctField and LIG perform slightly worse, with mIoU scores of 87.96 and 86.28, and F1 scores of 0.94 and 0.93, respectively. OccNet performs the worst, with an mIoU of only 71.36 and an F1 score of 0.70. IM-Net has an mIoU of 79.98 and an F1 score of 0.83, both lower than the proposed method. This indicates that the proposed method has a significant advantage in overall performance and can more accurately reconstruct shapes.

Table 2 provides a detailed comparison of the CD and EMD scores for different methods across five categories (Plane, Car, Chair, Table, and Sofa). The proposed method achieves the lowest scores in all categories, with an average CD of 2.79 and an average EMD of 1.94, demonstrating its superior performance in detail reconstruction and overall consistency. Specifically, the proposed method has the lowest CD and EMD scores in each category, such as a CD score of 2.06 and an EMD score of 2.33 in the Plane category, and a CD score of 2.34 and an EMD score of 1.49 in the Table category. OctField and LIG also perform well on these metrics, with average CD scores of 2.97 and 3.27, and average EMD scores of 2.19 and 2.63, respectively, but they still fall short of the proposed method. IM-Net and OccNet perform relatively poorly, particularly in terms of CD and EMD scores, with their average scores being higher than those of the proposed method.

Taken together, the method in this paper performs well on all assessment metrics, and not only has a significant advantage in overall performance, but also demonstrates excellent ability in detail reconstruction and consistency. This indicates that the method in this paper is highly stable and superior in the form reconstruction task, and is able to achieve the best performance in various categories and on different evaluation metrics. In contrast, other methods, although also performing better on some metrics, are still not as good as this paper’s method overall, especially OccNet and IM-Net, which underperform on a number of metrics.

### 4.2. Shape Generation and Interpolation

**Figure 6:**
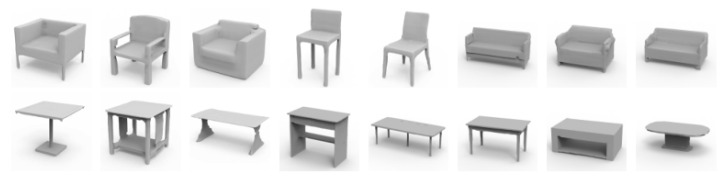
Shape generation. The image shows the results generated by randomly sampling potential codes in the potential space.

This method employs a GAN to train the network, which enables the model to generate various 3D shapes by randomly extracting normally distributed random noise vectors from the pre-trained decoder. The technique selects a potential vector at random from the learned potential space, decodes it into the shape space using Marching Cubes to extract its zero-point equivalent surface, and then creates a new three-dimensional shape. Figure 6 depicts the respective generation results for the chair and table categories. The current method can produce high-quality 3D objects with intricate geometric details despite using random sampling.

**Figure 7:**
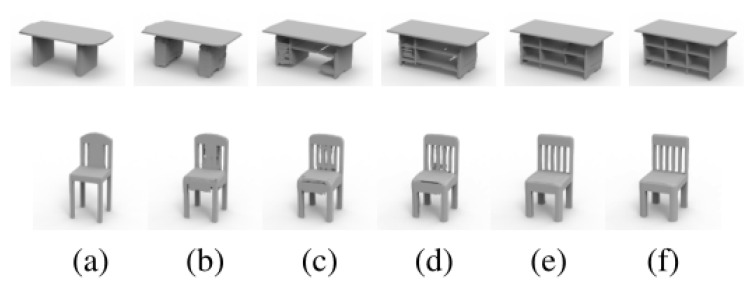
Shape interpolation. The figure shows the results of two types of interpolation: table and chair. (**a**) The source shape and (**f**) the target shape. (**b**–**e**) is the intermediate result of interpolation.

As a second technique for generating 3D models, interpolation operations can be performed on the provided shapes in the latent space. By linearly interpolating the latent codes of the two input shapes, the resulting latent code vectors are sent to a pre-trained decoder for shape interpolation. Figure 7 shows the interpolation results for the chair and table categories. This method enables continuous smooth interpolation between two models with different structures and heights. Additionally, during interpolation, narrow geometric features, such as the grid base of the table in the first row, can also be properly preserved. In three-dimensional modeling methodologies, shape generation and interpolation play pivotal roles. Shape generation refers to the process of constructing the target object in 3D space, while interpolation techniques are utilized to generate new data points between existing data, facilitating smooth transitions or the creation of new forms. Shape generation typically serves as the foundation of 3D modeling, determining the basic geometric morphology of the model. Interpolation techniques, on the other hand, build upon shape generation, assisting in fine-tuning model details and altering forms to better align with design requirements or the structural characteristics of real-world objects. This technology is particularly crucial when constructing complex biomimetic structures, as it effectively simulates the continuous variations and gradual changes found in nature, thereby enabling precise reproduction of natural forms.

## 5. Conclusions

This work proposes a novel hierarchical 3D surface implicit representation method called Sea-OctField. This method utilizes sparse voxel octree representation to construct adaptive local support regions around the target surfaces of interest. Sea-OctField not only efficiently captures the geometric structures of surfaces, but also demonstrates superior performance and effectiveness over traditional control methods in various shape modeling, reconstruction, and editing tasks. The introduction of this technique opens up new possibilities in the field of biomimetics, particularly in the design and optimization of biomimetic devices.

In biomimetics, the application of Sea-OctField can deepen our understanding of the complex structures and forms of surfaces found in nature. For example, it can be used to model and analyze the following biological structures: the scales or shells of marine organisms, which have highly intricate surface structures; the microtextures of plant leaves, which play a critical role in photosynthesis; and the surface morphology of insect wings, which enhance flight performance through specific geometric features. By modeling and analyzing these natural structures, Sea-OctField can provide novel insights and approaches for the design and optimization of biomimetic devices. These devices, including biomimetic submarines, underwater robots, and medical devices, can mimic specific structures and behaviors from nature, thereby enhancing their adaptability and efficiency in fields such as marine exploration, environmental monitoring, and medical treatment.

Future work will focus on further leveraging the Sea-OctField method to advance biomimetics and biomimetic device development. This may involve refining the algorithms and performance of Sea-OctField to meet the demands for efficiency and sustainability of biomimetic devices. Additionally, integrating semantic information into the octree structure can enhance Sea-OctField’s ability to simulate and understand biological structures in nature, providing more inspiration and guidance for the design and optimization of biomimetic devices. These efforts will drive the advancement of the field of biomimetics and foster the widespread application of biomimetic devices across various domains.

## Figures and Tables

**Table 1:** Here, the mIoU and F1 scores are provided for quantitative evaluation of the formal reconstruction.

Method	IM-Net	OccNet	LIG	Octfield	Ours
mIoU	79.98	71.36	86.28	87.96	**88.32**
F1	0.83	0.70	0.93	0.94	**0.95**

**Table 2:** Evaluation quantitative of form reconstruction. The scores (lower is better) for the five categories CD ($10^{-4}$) and EMD ($10^{-2}$) are displayed in this table. Sea-OctField is compared to four baselines (IM-Net, OccNet, Local Implicit Grids, and OctField) in order to determine the average score and highest performance in each category.

Method		Plane	Car	Chair	Table	Sofa	Mean
IM-Net	CD	4.21	15.14	6.99	8.03	7.95	8.46
	EMD	3.39	4.46	3.77	3.16	2.51	3.45
OccNet	CD	5.62	13.54	7.87	7.47	8.6	8.62
	EMD	3.46	4.93	4.16	3.34	2.81	3.74
LIG	CD	2.50	5.46	2.37	2.81	3.23	3.27
	EMD	2.57	4.08	2.18	2.27	2.06	2.63
OctField	CD	2.29	4.84	2.19	2.53	3.02	2.97
	EMD	2.47	2.79	2.13	1.71	1.84	2.19
Ours	CD	**2.06**	**4.65**	**2.01**	**2.34**	**2.89**	**2.79**
	EMD	**2.33**	**2.31**	**2.02**	**1.49**	**1.54**	**1.94**

## Data Availability

The datasets presented in this article are not readily available because the data are part of an ongoing study. Requests to access the datasets should be directed to luyunhong@ytu.edu.cn.
